# MuscleAtlasExplorer: a web service for studying gene expression in human skeletal muscle

**DOI:** 10.1093/database/baaa111

**Published:** 2020-12-18

**Authors:** Olof Asplund, Johan Rung, Leif Groop, Rashmi Prasad B, Ola Hansson

**Affiliations:** Genomics, Diabetes and Endocrinology Unit, Department of Clinical Sciences, Lund University Diabetes Centre, Jan Waldenströms gata 35, Malmö 20502, Sweden; SciLifeLab, BMC, Husargatan 3, Uppsala University, Uppsala 751 22, Sweden; Genomics, Diabetes and Endocrinology Unit, Department of Clinical Sciences, Lund University Diabetes Centre, Jan Waldenströms gata 35, Malmö 20502, Sweden; Institute for Molecular Medicine Finland (FIMM), University of Helsinki, Tukholmankatu 800290 Helsinki, Finland; Genomics, Diabetes and Endocrinology Unit, Department of Clinical Sciences, Lund University Diabetes Centre, Jan Waldenströms gata 35, Malmö 20502, Sweden; Genomics, Diabetes and Endocrinology Unit, Department of Clinical Sciences, Lund University Diabetes Centre, Jan Waldenströms gata 35, Malmö 20502, Sweden; Institute for Molecular Medicine Finland (FIMM), University of Helsinki, Tukholmankatu 800290 Helsinki, Finland

## Abstract

MuscleAtlasExplorer is a freely available web application that allows for the exploration of gene expression data from human skeletal muscle. It draws from an extensive publicly available dataset of 1654 skeletal muscle expression microarray samples. Detailed, manually curated, patient phenotype data, with information such as age, sex, BMI and disease status, are combined with skeletal muscle gene expression to provide insights into gene function in skeletal muscle. It aims to facilitate easy exploration of the data using powerful data visualization functions, while allowing for sample selection, in-depth inspection and further analysis using external tools.

**Availability:**

MuscleAtlasExplorer is available at https://mae.crc.med.lu.se/mae2.

## Introduction

Microarrays have been widely used for analyzing gene expression in biological samples. More recently, RNA sequencing (RNA-Seq) has become the prevailing technology, but is still limited by high running costs compared to array-based platforms.

Since the introduction of microarrays more than 20 years ago (1), a large amount of raw data have been archived in public databases. As of January 2020, there are almost three times more human gene expression samples from microarrays (970 530) in the Gene Expression Omnibus than from RNA-Seq data (333 074). Combination of these data across studies would allow for the analysis of a very large number of samples without any new experimental cost. A prerequisite is, of course, that the phenotype data is highly structured and detailed. To enable this type of analysis, Su *et al.* (2) manually curated the phenotypic information from 44 microarray studies of human skeletal muscle, allowing analysis of all samples as a single dataset. This dataset (European Bioinformatics Institute (EBI) project E-MTAB-1788) (3) contains expression data from 19 603 genes from a total of 1654 samples connected with 40 different phenotypes.

The dataset has already been utilized in several ways (4–24). Here we make it even more accessible by the creation of MuscleAtlasExplorer (MAE). This web service facilitates fast lookups of gene expression using a simple and powerful interface, which allows for analyses that can go far beyond the scope of the original article.

## Materials and methods

### Data preprocessing

The dataset consists of two parts. The patient metadata contains the phenotypes of individuals, such as age, gender and BMI, as well as experimental protocol information. To make the application easy to use, we have limited the available metadata to a set of 16 variables deemed to be non-redundant and particularly relevant.

The expression data contain information on the relative amount of expression from a single normalized analysis for each sample and gene (2). The curated dataset has data from two major microarray platforms, i.e. Affymetrix ‘HGU133Plus2’ and ‘HGU133-A’. The results from each platform are plotted and presented separately to avoid platform-specific biases.

### Web interface

Computations and plotting are performed using R statistical software (25) and ggplot2 package (26). The Shiny framework (27) provides a graphical interface in the browser of the user.

## Results

The application has four major parts: (i) a gene lookup view that gives a quick overview of a chosen gene, (ii) a phenotype view that focuses on phenotype data, (iii) a gene-centric view that focuses on the gene expression data and (iv) an association view with focus on associations between phenotypes and gene expression.

### Gene lookup

**Figure 1.**
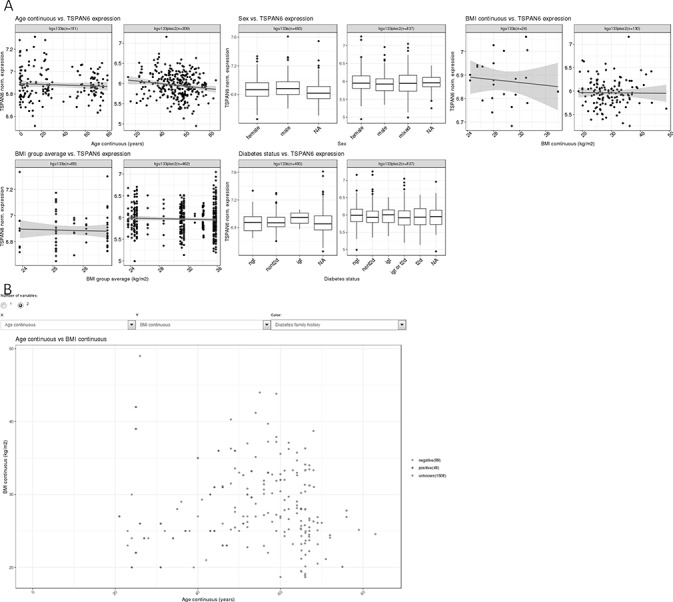
(A) Example of the gene summary view, showing expression data for the gene H3 histone, family 3B (H3F3B) in relation to important phenotypes. (B) One example of two-variable plots produced by MuscleAtlasExplorer, i.e. scatter plots for two different continuous variables (exemplified by age and BMI, with coloring set to signify diabetes family history).

The user can search for information regarding a specific gene in the Gene Lookup view. Upon selection of the gene,a short summary of associations between phenotypes and gene expression is shown for each phenotype including plots. The gene glycogen synthase 1 is shown as an example in Figure 1A.

### Phenotypes

MAE has several functions to characterize and select patients. The Phenotype tab allows users to filter patients based on phenotype. Filters applied in this tab are used in most other parts of the web application. The table of patients can be exported as a comma-separated file, for further analysis using other programs.

The ‘visualize samples’ tab allows the user to investigate phenotypes in more detail. The phenotypes can be visualized either using a table or using different plotting functions.

The ‘Custom Plot’ (Figure 1B) function allows the user to plot any phenotype.

Two different phenotypes can also be plotted in relation to each other. This allows for more detailed analysis. In addition, each data point can be colored depending on a third phenotype.

### Genes

The ‘Genes’ view show data on gene expression. Users can search for genes of interest using symbols, Ensembl IDs or trivial names. Clicking a gene in this table will select that gene for plotting under the ‘Gene plots’ tab. Only patients that have been previously selected under the ‘Samples’ view are shown.

### Associations

The ‘Associations’ view allows users to explore the relationship between gene expression and phenotypes. Five linear models have been precomputed, using a similar methodology as in Su *et al*. (2), to identify genes that are differentially expressed in relation to age, gender, type 2 diabetes status, acute training and BMI.

## Discussion

Data repositories such as Gene Expression Omnibus (28,29), ArrayExpress (30,31) and Sequence Read Archive (32) provide massive amounts of raw data, but the phenotypes are not easy to standardize. As a result, these services have limited analysis capabilities within the websites. Services such as Genotype Tissue Expression project (GTEx) (33) and Ensembl (34) provide tools for analysis, but do not contain detailed phenotype data. Skeletal muscle–specific tools and datasets are also available. Examples of this include MetaMEx (35), which gives detailed information regarding gene expression in association with intervention studies as well as gene–gene correlations in skeletal muscle. NeuroMuscleDB (36) provides a database of genes active during different stages of muscular development, and SysMyo (37) provides gene sets of genes associated with a vast variety of neuromuscular conditions and experimental intervention studies. We hope that MAE will work in a complementary manner for these datasets, providing in-detail visualization of skeletal muscle gene expression. For this dataset (2), we have leveraged the highly structured metadata to provide information on associations between gene expression and phenotypes. This way, we hope to augment the wealth of information from other databases with functional association data in regard to skeletal muscle gene expression.

The Shiny framework was selected for development because it allows the developer to leverage the powerful plotting and processing capabilities of R, while providing an easy-to-use interface.

MAE bases its plots only on microarray gene expression data. In contrast, in the modern day, high-throughput sequencing is the prevailing technology for gene expression analysis. There are some technical disadvantages to microarrays for gene expression analysis. For instance, the restriction of gene coverage to the targeted probe regions can decrease the sensitivity to detect certain genes. Furthermore, the nature of microarray technology can decrease the sensitivity to detect genes with low expression. On the other hand, the much lower cost of microarrays and vast availability of open datasets increase the power to detect differentially expressed genes.

It is expected that users will need to extend the analysis using external software. For this reason, we have included functions to export the relevant data. The openly available source code, available at https://github.com/olof-a-bio/muscle-atlas-explorer, allows users to extend and modify the plotting and analysis functions in the web application for their own use.

We thereby consider the MAE as a new resource to facilitate research needing information on gene expression in human skeletal muscle.
